# Cancer staging at diagnosis data comparisons in South Australia

**DOI:** 10.1038/s41598-020-57704-5

**Published:** 2020-01-23

**Authors:** Rosie Meng, Kamalesh Venugopal, Helen Thomas, Katina D’Onise

**Affiliations:** Prevention and Population Health Directorate, Wellbeing SA, Government of South Australia, Adelaide, South Australia Australia

**Keywords:** Cancer epidemiology, Cancer epidemiology

## Abstract

Cancer stage at diagnosis is an important gap for Australian population based cancer registries. The study aims to understand the quality and completeness of three different collections of cancer staging data. The South Australian Cancer Registry data collection for breast and colorectal cancer (CRC) cases diagnosed in 2011, was linked to Registry Derived Stage (RDS) data, pathology plus hospital metastasis codes (pathology stage), and the South Australian Clinical Cancer Registry Stage (SACCR stage). The agreement between staging systems was examined using kappa statistics. Kaplan-Meier curves and Cox regression were used to examine the difference in survival by staging methods. Among 2,530 breast and CRC cases 98.8% were stageable (n = 2,500) according to histology. Among stageable cases, 84.6% had RDS, 51.2% had pathology stage and 29.5% had SACCR stage. The kappa statistic for RDS and pathology stage was 0.930 for breast cancer and 0.973 for CRC, and 0.574 for RDS and SACCR stage for breast cancer and 0.632 for CRC. The agreement between pathology stage and SACCR stage was 0.430 for breast cancer and 0.528 for CRC. The distribution of stage was similar across staging methods, although more stage four cancers by pathology stage, and survival patterns were similar but not the same. The agreement was high between different staging systems. Pathology stage had a higher than expected stage 4 proportion. This study highlights an opportunity to collect stage information in a cost-effective manner, while collecting data that usefully represent stage at diagnosis across the population, for population based epidemiological analyses.

## Introduction

Cancer, as a collective, is now the leading cause of burden of disease in Australia, accounting for 19% of the total burden^1^. While rates of cancer diagnosis are declining, the number of diagnoses continues to rise and so there is an ongoing need to better understand the population dynamics of cancer over time for health systems. Cancer registries in Australia have been collecting data on the incidence and mortality of invasive cancer since as early as 1972, providing highly valuable data to inform health service planning, prevention policy, the evaluation of interventions and for research purposes^2^. Despite the value presented in population based cancer registries^3,4^, there is a clear need to expand them to include stage at diagnosis^5^. From an epidemiological perspective, such information allows for a more complete analysis of trends, and in particular allows for a more thorough understanding of potentially causal factors for underlying shifts in incidence and mortality over time. This information is invaluable to population health, including cancer screening, health care services and for comparisons with other jurisdictions^6^.

There are however challenges in collecting valid, reliable and complete data on stage at diagnosis at the population level^7,8^. Information in Australia is notified under legislation to population based cancer registries from a variety of sources, but these notifications generally do not include detailed clinical information on which to make an assessment of stage at diagnosis. Further, collecting stage at diagnosis as an additional data item in the setting of increasing cancer notifications represents a challenge in resource constrained environments. As such, it is important to evaluate the quality and completeness of stage data collected at the population level, in addition to considering the resource implications of the different staging collection systems. This evaluation will be necessary to understand the strengths and limitations of the staging data and determine how the data should be used in an ongoing way.

South Australia was a participant in the ‘National Collection of Registry Derived Stage for 2011’ project. This project, led by Cancer Australia, examined the feasibility of Australian population based cancer registries meeting epidemiological demands for comparability and completeness in collecting disease stage at diagnosis for all eligible breast, lung, colorectal, melanoma and prostate cancers diagnosed in 2011 across Australia^9,10^. These data present an opportunity to evaluate the completeness and quality of the systematically collected population based staging data against other state based cancer staging systems in South Australia to better understand the value of the data collected from population based staging systems going forward.

## Methods

### Data

There are four main data sources for this study: the South Australian Cancer Registry (SACR), Registry Derived Stage (RDS), pathology stage and the South Australian Clinical Cancer Registry (SACCR).

The SACR has been in operation since 1977, collecting incidence and mortality data on all invasive cancers^11^. The notification sources, under legislation, are pathology, hospital admissions, deaths from the state based Births, Deaths and Marriages registry, in addition to radiotherapy notifications.

The data collected under the ‘National Collection of Registry Derived Stage for 2011’ project, hereafter referred to as RDS at diagnosis, used an agreed set of coding rules for staging at the population level, endorsed by the Australasian Association of Cancer Registries. The data were collected for all cases from the top five high incidence cancers (prostate, breast, lung, bowel, melanoma) in the SACR diagnosed in 2011. The sources of data used for this project were copies of all relevant pathology reports, summary stage data from radiotherapy notifications and summary information on metastatic disease supplied by hospital health information systems. RDS was assigned by coding staff experienced in staging data according to the AJCC 7 rules. The AJCC staging system is a classification system developed by the American Joint Committee of Cancer for describing the extent of disease progression in cancer patients^12^. Within the RDS system, a case was coded as “unknown stage” when the staging elements that are necessary for determining stage were incomplete. RDS was not derived for cases where the basis of diagnosis was solely clinical or the case was described as a death certificate only, as there was no pathology in these instances. Ten percent of all RDS cases were double-coded entirely by a second person to ensure reliability across coding staff. RDS was defined as the best estimate of summary TNM stage at the time of diagnosis from available data sources for use in population-based cancer registries^9^.

Data were extracted from the SACR for all eligible cases diagnosed in 2011 and pathology reports were collated for each case. Summary data from the SACR, including stage at radiotherapy were collated into a purpose built database where RDS was entered by coding staff. Coding rules for each of the primary sites being staged were supplied to the coding staff along with training and ongoing support in the use of the staging system. At the completion of the coding exercise, data were extracted into a file for analysis.

In April 2018, the cancer stage at diagnosis project was complete, and the project found that staging completeness and comparability across Australia was high for each of the top five incidence cancers^9^. The current study used the data only for South Australian breast and colorectal cancer (CRC) due to the availability of clinical cancer registry staged data as a comparison, and further that both breast and CRC are screened for in the national population based screening program.

The second source of staging data was the TNM (AJCC7) information as was reported on the pathology report, where available. Given pathology alone may miss metastatic disease, this information was combined with a notification in the hospital admissions data of a metastatic cancer code. Hospital admission data containing a cancer code in the ICD-10 range of C78-C79 inclusive, within four months of the first diagnosis date of the cancer were flagged as metastatic disease. Where there was a metastasis notified as above, the summary stage was recorded as a stage 4. This data source has been referred to as pathology stage.

The third source of staging data was the SACCR^13^. The SACCR is authorised under the *Health Care Act 2008*^14^, and covers a range of cancer types relating to admissions to one of four public hospitals in metropolitan Adelaide. The SACCR is supported by a central coordinating unit, and uses the full medical record as the source of information about the cancer case. The SACCR uses the AJCC 7 staging system for both breast and colorectal cancer. For each of these cancers, the SACCR has registered around one third of the total South Australian cases in 2011. This staging source is referred to as SACCR stage.

### Data linkage

The RDS data and pathology stage were already linked to the SACR. The SACCR data were matched to the SACR using a common unique identification number. For cases that were not matched, patient’s name, date of birth, sex, and cancer were used in a probabilistic linkage. After completing data linkage, data were de-identified and provided to a separate team for data analysis.

### Statistical method

A descriptive analysis was undertaken, comparing the demographics and staging information and completeness across the three staging systems (RDS, SACCR stage and pathology stage). The degree of agreement was evaluated using the Cohen’s kappa score. This analysis only used the matched data set for each of breast and colorectal cancer. Kappa agreement classification is as following: poor (<0.20), fair (0.20–0.40), moderate (0.40–0.60), good (0.60–0.80), and very good (0.80–1.00). Using all the available data (both matched and non-matched data), Kaplan-Meier survival estimates were used to examine whether there was any inconsistency for five year survival by the different staging systems. The censor date was the date of death or 31 December 2016, whichever came first. The cause-specific death information was taken from the SACR dataset given it was complete for all cases. The Cox proportional hazard model was used for examining cancer cause-specific mortality across the three staging methods. All analyses were performed using Stata SE 15.0 (Stata Corp, Texas).

All methods were performed in accordance with the relevant guidelines and regulations of Scientific Reports. The study is a retrospective data linkage project using existing data to evaluate registry data quality, and there was no any patient contact for the study, therefore there was no patient consent process. The Human Research Ethics Committee, South Department for Health and Ageing approved the project in December 2017. The ethics commit waived the need for informed consent for this study as part of the study approval.

## Results

### Completeness of staging data for sources

There were 2,530 breast and colorectal cancer cases in the SACR 2011 data collection. Based on histology, 98.8% were stageable (n = 2,500). Among cases stageable on the basis of histology, 84.5% (n = 2,115) had RDS, 51.2% (n = 1,279) had pathology staging values, and 29.5% (n = 737) had SACCR stage values. Breast cancer had staging information on 88.1% of cases with RDS, and colorectal had stage information for 81.3%. The proportions were similar for breast and colorectal cancer for pathology stage (51.5% vs 50.8%) and SACCR stage (29.6% vs 27.8%. The details of the demographics across the three data sources are presented in Table 1. The proportion of unknown or missing RDS was 11.9% for breast cancer (n = 143) and 18.5% for CRC (n = 242). The proportion of unknown or missing SACCR stage was 7.1% for breast cancer (n = 26) and 3.2% for CRC (n = 12). Among available cases with pathology stage values, there was no unknown or missing value for either breast cancer or CRC.

**Table 1:** Demographic and staging information for breast cancer, from a total number of stageable cases of 1204 South Australia, 2011.

Characteristics	RDS		Pathology stage		SACCR Stage^a^		P value^b^
	n	%	n	%		%	
**Breast cancer**	1204	100.0	620	100.0	364	100.0	
Sex							0.73
Male	8	0.7	2	0.3	2	0.5	
Female	1,196	99.3	618	99.7	362	99.5	
Age group year							0.11
<40	37	3.1	21	3.4	16	4.4	
40–49	180	15.0	105	16.9	64	17.6	
50–59	250	20.8	134	21.6	76	20.9	
60–69	337	28.0	192	31.0	89	24.4	
70–74	128	10.6	68	11.0	44	12.1	
≥75	272	22.6	100	16.1	75	20.6	
SEIFA	n = 1203		n = 619		n = 363		<0.001
Quintile 1 most disadvantaged	204	17.0	97	15.7	98	27.0	
Quintile 2	225	18.7	114	18.4	69	19.0	
Quintile 3	223	18.5	118	19.1	72	19.8	
Quintile 4	267	22.2	130	21.0	63	17.4	
Quintile 5 least disadvantaged	284	23.6	160	25.8	61	16.8	
Stage (unknown/missing excluded^c^)	n = 1061		n = 620		n = 356		
1	527	49.7	276	44.5	111	31.2	
2	319	30.1	192	31.0	150	42.1	
3	87	8.2	42	6.8	69	19.4	
4	128	12.1	110	17.7	26	7.3	
**Colorectal cancer**	1296	100.0	659	100.0	373	100.0	
Sex							0.73
Male	696	53.7	360	54.6	223	59.8	
Female	600	46.3	299	45.4	150	40.2	
Age group year							0.11
<40	15	1.2	7	1.1	8	2.1	
40–49	49	3.8	29	4.4	23	6.2	
50–59	173	13.3	100	15.2	64	17.2	
60–69	288	22.2	152	23.1	88	23.6	
70–74	185	14.3	98	14.9	45	12.1	
≥75	586	45.2	273	41.4	145	38.9	
SEIFA	n = 1294		n = 659		n = 372		<0.001
Quintile 1 most disadvantaged	259	20.0	115	17.5	96	25.7	
Quintile 2	303	23.4	144	21.9	90	24.1	
Quintile 3	258	19.9	146	22.2	79	21.2	
Quintile 4	249	19.2	130	19.7	56	15.0	
Quintile 5 least disadvantaged	225	17.4	124	18.8	51	13.7	
Stage (unknown/missing excluded^c^)	n = 1,054		n = 659		n = 360		
1	219	20.8	68	10.3	36	10.0	
2	308	29.2	155	23.5	85	23.6	
3	236	22.4	150	22.8	136	37.8	
4	291	27.6	286	43.4	103	28.6	

^a^Data are from the SACCR with the cases that are matched to the SACR. There were 7 breast cancer and 11 colorectal cancer patients with diagnoses of two primary sites, and they are counted twice in analyses.

^b^P values are derived from chi-square test or Fisher exact test wherever is appropriate.

^c^Breast cancer: 11.9% unknown/missing RDS (n = 143), and 7.1% unknown/missing SACCR Stage (n = 26). Colorectal cancer: 18.5% unknown/missing RDS (n = 242), and 3.2% unknown/missing SACCR Stage (n = 12).

### Agreement across three staging methods

There were no significant differences in the age and sex distribution for breast or colorectal cancer across three staging methods (all p > 0.05). However, the SACCR staging data had a higher proportion of people in the lowest quintile of SEIFA (27% most disadvantaged) than the RDS data and the pathological stage data (17% and 15.7% respectively).

There were 619 breast and 652 CRC cases for which there was a matched cancer case with the RDS and pathology stage data. The agreement between RDS and pathology stage was very good, with a kappa statistic of 0.930 for breast cancer and 0.973 for CRC (Table 2). There were 331 breast cancer and 317 CRC cases for which there was a matched cancer case with the RDS and SACCR stage data. The agreement between RDS and SACCR stage was good for breast cancer (kappa = 0.574) and CRC (kappa = 0.632). There were 162 breast cancer and 205 CRC cases for which there was a matched cancer case with the pathology stage and SACCR data. The agreement between pathology stage and SACCR stage was moderate for breast cancer (kappa = 0.430) and good for CRC (kappa = 0.528).

**Table 2:** Agreement between the different staging methods (RD Stage, Pathological Stage and Clinical Stage), South Australia, 2011.

Cancer type	Statistics	RDS vs. Pathology stage	Pathology stage vs. SACCR stage	RDS vs. SACCR Stage
**Breast**		n = 619	n = 162	n = 331
	Number of observed agreements	590 (95.3%)	90 (55.6%)	229 (69.2%)
	Number of agreements expected by chance	205 (33.1%)	36 (22.0%)	91 (27.6%)
	**Kappa**	**0.930**	**0.430**	**0.574**
	95% CI of Kappa	0.911–0.940	0.343–0.510	0.543–0.645
	Strength of agreement using Kappa	Very good	Moderate	Moderate
	Weighted Kappa	0.956	0.484	0.625
	Strength of agreement using weighted Kappa	Very good	Moderate	Good
**Colorectal**		n = 652	n = 205	n = 317
	Number of observed agreements	640 (98.2%)	143 (69.8%)	233 (73.5%)
	Number of agreements expected by chance	200 (30.6%)	74 (35.9%)	88 (27.9%)
	**Kappa**	**0.973**	**0.528**	**0.632**
	95% CI of Kappa	0.958–0.980	0.460–0.550	0.596–0.653
	Strength of agreement using Kappa	Very good	Moderate	Good
	Weighted Kappa	0.977	0.648	0.728
	Strength of agreement using weighted Kappa	Very good	Good	Good

Note: RDS values are from the RD Stage project dataset, Pathology stage values are from the SACR, and SACCR stage values are from the SACCR. Only include cases with stage 1–4 in the analysis.

### Staging distribution comparison across staging data sources

**Figure 1:**
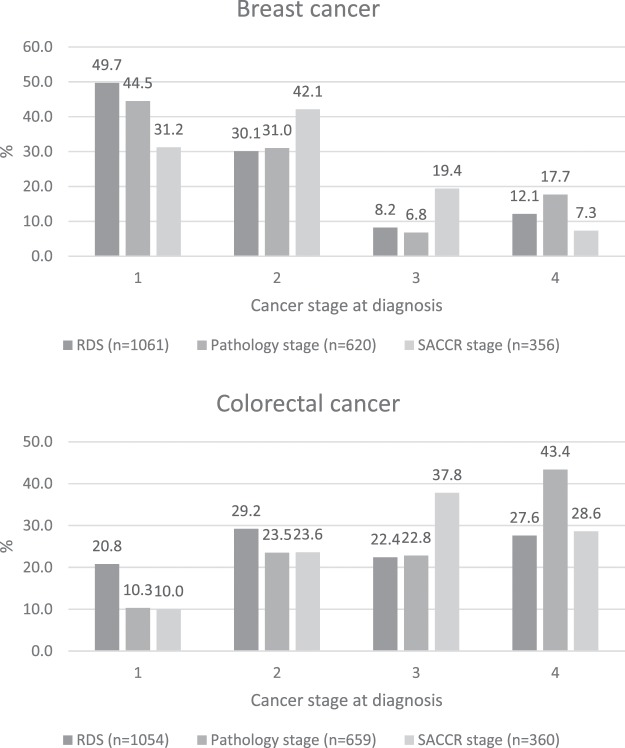
Staging distribution comparison across RDS, pathology stage and SACCR stage, breast cancer (n = 1061) & colorectal cancer (n = 1054), South Australia, 2011.

When examining all available data, that is both data that were matched across the different staging systems and non-matched data, pathology stage has a significantly higher proportion of stage 4 cases when compared to RDS (mean difference in proportion = 5.6%, 95%CI = 2.0–9.2%, p = 0.002) and SACCR stage (mean difference in proportion = 10.4%, 95%CI = 6.4–14.4%, p < 0.001) for breast cancer (Fig. 1).

A similar pattern was observed for colorectal cancer, with pathology stage having a significantly higher proportion of stage 4 cases when compared to RDS (mean difference in proportion = 15.8%, 95% CI = 11.1–20.4%) and SACCR stage (mean difference in proportion = 3.1%, 95% CI = 8.8–20.8%, all p < 0.001, Fig. 1).

### Cancer cause-specific survival comparison across staging data sources

**Figure 2:**
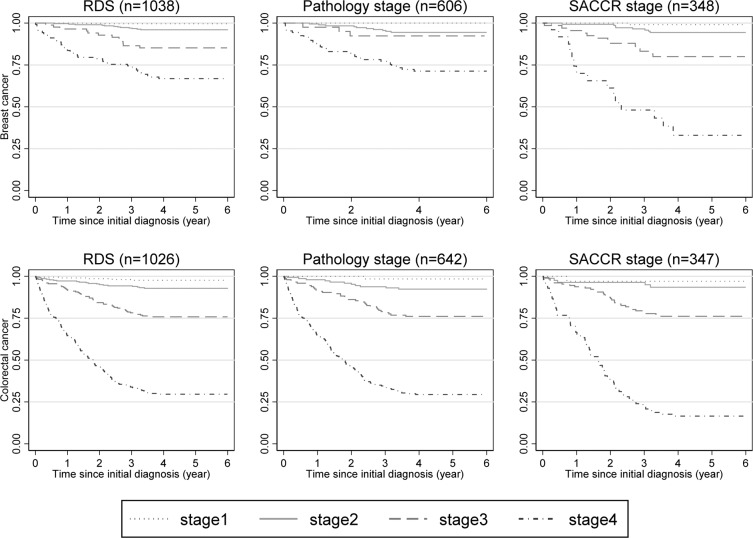
Kaplan-Meier survival estimates comparison by using different staging methods, breast cancer and colorectal cancer cause-specific, South Australia, 2011. Log rank test was performed for all staging methods and all p values are < 0.01.

Using all available data, the distribution of survival by stage was similar by three staging methods for breast cancer and colorectal cancer (Fig. 2). The one year survival for stage 4 breast and colorectal cancer cases were similar across three staging methods, 70–87% for breast cancer and 64–67% for CRC. However, a more distinguished worse survival in stage 4 cases was observed using SACCR stage in both breast cancer and CRC. The five year survival for stage 4 breast cancer was 33.0% when using SACCR stage, but was 66.9% by RDS and 71.4% by pathology stage. The five year survival for stage 4 CRC was only 16.5% using SACCR stage, but was 29.6% by RDS and 29.4% by pathology stage.

The Cox proportional hazard models for breast cancer and CRC by all three staging methods found similar results across the three staging methods for both types of cancer (Table 3). For breast cancer, stage 4 cases had a significantly greater risk of dying from breast cancer than stage 1 or 2 cases by each of the three staging methods. For CRC, stage 3 or 4 cases had significantly greater risk of dying from CRC than stage 1 or 2 cases.

**Table 3:** Number of cause-specific death and hazard ratio by different staging methods.

	RDS	Pathology stage	SACCR stage
**Breast cancer**			
**Number of cause-specific death/total number of patients in each stage**			
Stage	n = 1038	n = 606	n = 348
1	2/515 (0.4%)	0/269 (0%)	1/108 (0.9%)
2	12/311 (3.9%)	10/188 (5.3%)	8/146 (5.5%)
3	12/86 (14.0%)	3/42 (7.1%)	13/69 (18.8%)
4	41/126 (32.5%)	30/107 (28.0%)	15/25 (60/0%)
**Hazard ratio & 95% CI from Cox regression models**			
Stage			
1	0.10 [0.02, 0.44]^**^	0.00	0.17 [0.02,1.33]
2	1.00 (reference)	1.00 (reference)	1.00 (reference)
3	3.95 [1.77, 8.79]^***^	1.43 [0.39, 5.20]	3.97 [1.65, 9.59]^**^
4	10.27 [5.39, 19.59]^***^	6.10 [2.98, 12.49]^***^	18.73 [7.91, 44.38]^***^
**Colorectal cancer**			
**Number of cause-specific death/total number of patients in each stage**			
Stage	n = 1026	n = 642	n = 347
1	5/217 (2.3%)	1/67 (1.5%)	1/34 (2.9%)
2	20/299 (6.7%)	11/154 (7.1%)	5/84 (5.9%)
3	53/229 (23.1%)	34/147 (23.1%)	30/129 (23.3%)
4	185/281 (65.8%)	182/274 (66.4%)	81/100 (81.0%)
**Hazard ratio & 95% CI from Cox regression models**			
Stage			
1	0.32 [0.12, 0.86]^*^	0.19 [0.03, 1.51]	0.45 [0.05, 3.83]
2	1.00 (reference)	1.00 (reference)	1.00 (reference)
3	3.66 [2.19, 6.12]^***^	3.41 [1.73, 6.73]^***^	3.95 [1.53, 10.18]^**^
4	16.38 [10.30, 26.04]^***^	15.70 [8.53, 28.91]^***^	25.44 [10.27, 63.00]^***^

*p < 0.05; ^**^p < 0.01; ^***^p < 0.001.

## Discussion

This study found that staging completeness overall was very high for both breast and colorectal incidence cancers at diagnosis in the RDS data, and lowest for the SACCR stage data as SACCR only collects public hospital data. The different staging systems had similar age and sex profiles, but the SACCR included more people from the most disadvantaged groups. There was a good to very good agreement for both breast and colorectal cancer for each of the data sources when considering only the matched data, and while they each had a similar profile of stage at diagnosis across the population, the pathology stage had proportionately higher stage 4 cases than the other two. This difference can be explained by the pathology stage data in its entirety provides a different stage profile than does the pathology data that is matched to the other data sources. The different staging systems were equally able to discriminate survival by stage, although the actual survival estimates were different from each other. It is likely, however, that the differences found between the staging systems still present a reasonable estimate of stage at diagnosis at the population level (for epidemiological analyses), and in particular, that the two population based systems (pathology stage and RDS) are a reasonable estimate of the true distribution of stage at diagnosis at the population level.

The completeness of staging data for the South Australian RDS was 99.6% for breast cancer and 98.1% for CRC, which compared well with the Australian national average of 94% for female breast cancer and 88% for CRC^10^. The percentage of stage ‘unknown’ was 11.9% for breast cancer and 18.5% for CRC which is slightly higher than was reported in Canada for data from 2010, with 10% of unknown stage cases^15^.

The cancer staging distributions by RDS for breast cancer and CRC are similar to Australian national figures, which is that the majority of cancers were staged as an ‘early stage’^10^. Data from the Surveillance, Epidemiology, and End Results (SEER) in the United States from 2004 to 2011 demonstrated the proportion of breast cancer cases at diagnosis for stage I, II, III and IV were 48.0%, 34.6%, 12.4% and 5.0% respectively^16^. This is similar to the stage profile of the SACCR in this study, but the RDS and pathology stage of South Australia breast cancer 2011 collection has higher percentage of stage IV breast cancer (12.1% & 17.7% respectively) than the SEER’s. The outlier in the staging data was the higher proportion of stage 4 in the colorectal pathology data, despite the degree of agreement between this source and the other sources being high.

The difference between the high degree of agreement on matched data but not on stage at diagnosis profile across the population most likely reflects selection bias in those cases that had a pathology stage and those that had the other staging systems. Pathology stage is a relatively small sub-set of the total number of stageable cases, and so likely represents a degree of selection bias, biasing the proportion of stage 4 cases upward. The SACCR data are only collected from some public hospitals, and so would not be representative of a whole of population stage at diagnosis. Given the RDS system had the highest level of completeness, and estimates a similar stage at diagnosis profile as in other jurisdictions, it should be considered the most valid of the three systems for population level estimates. Over time, given the high degree of agreement with the RDS system, as completeness of the pathology data increases with increased focus on the importance of structured pathology reporting, this bias may lessen^17^.

The survival curves by the different staging systems for breast cancer and CRC were similar although not the same. For both breast cancer and CRC, the survival curve by the three staging methods all seemed to be consistent with the clinical expectation – better survival for cases diagnosed at early stage and survival decreased markedly with advancing stage. The SACCR stage pattern for both breast cancer and CRC are similar to those published in the United States for five-year survival^18,19^ and United Kingdom for one-year survival^20^. Data from the National Cancer Registration Services Cancer Analysis System in England in 2012 shows that the one year relative survival for stage 4 cases were 66.4% for breast cancer and 42.5% for CRC^20^, slightly lower than the current study using SACCR stage (breast cancer 70.0% and CRC 66.5%). For distant cancer cases using SEER stage, the American Cancer Society reported a 5-year relative survival rate of 27% for breast cancer and 15% for CRC^18,19^, which were very close to survival of stage 4 cases by the SACCR stage method in the current study (33.0% for breast cancer and 16.5% for CRC).

The strengths of this study include an opportunity to examine three different staging systems collected on the one group of cancer cases in a high quality, population based cancer registry. The data were coded by trained and experienced coding staff and a set of clear coding rules were applied across all systems. Limitations include that only one third of 2011 breast and colorectal cancer cases had the SACCR stage values, and about 50% had pathology stage, which may have introduced selection bias into the findings presented here. The population of cancer cases did however appear to be similar across the three methods, albeit that there were more people from a higher level of disadvantage in the SACCR data than the other two and that only three variables were available for demographic comparison. This study should be repeated over time to better understand how the data quality may change. In particular, pathology stage is likely to have a higher number of cases staged over time, and so it may be that the data quality and ascertainment is better for cases diagnosed in 2019 than when this study was set, in 2011.

## Conclusions

In conclusion, the data from the two population-based staging systems compared favourably with the clinical based staging system across a number of metrics. This represents an opportunity to collect stage information in a cost-effective manner, while collecting data that usefully represent stage at diagnosis across the population, for population based epidemiological analyses.

## Data Availability

The datasets analysed for the current study are not publicly available due to the restriction term in the ethics approval. The de-identified data could be available for the editorial team under a confidential agreement, however, for some variables, categories may be combined in order to reduce the risk of revealing patients’ identity incidentally.
